# Supplementary material for: A Global Screen for Assembly State Changes of the Mitotic Proteome by SEC-SWATH-MS
Source: Cell Syst. 2020 Feb 26;10(2):133–155.e6. doi: 10.1016/j.cels.2020.01.001 (PMC7042714; doi:10.1016/j.cels.2020.01.001)

P29034 | S10A2_HUMAN | S100A2 S100L
Monomer MW [kDa]: 11.117 Monomer expected elution fraction: 59

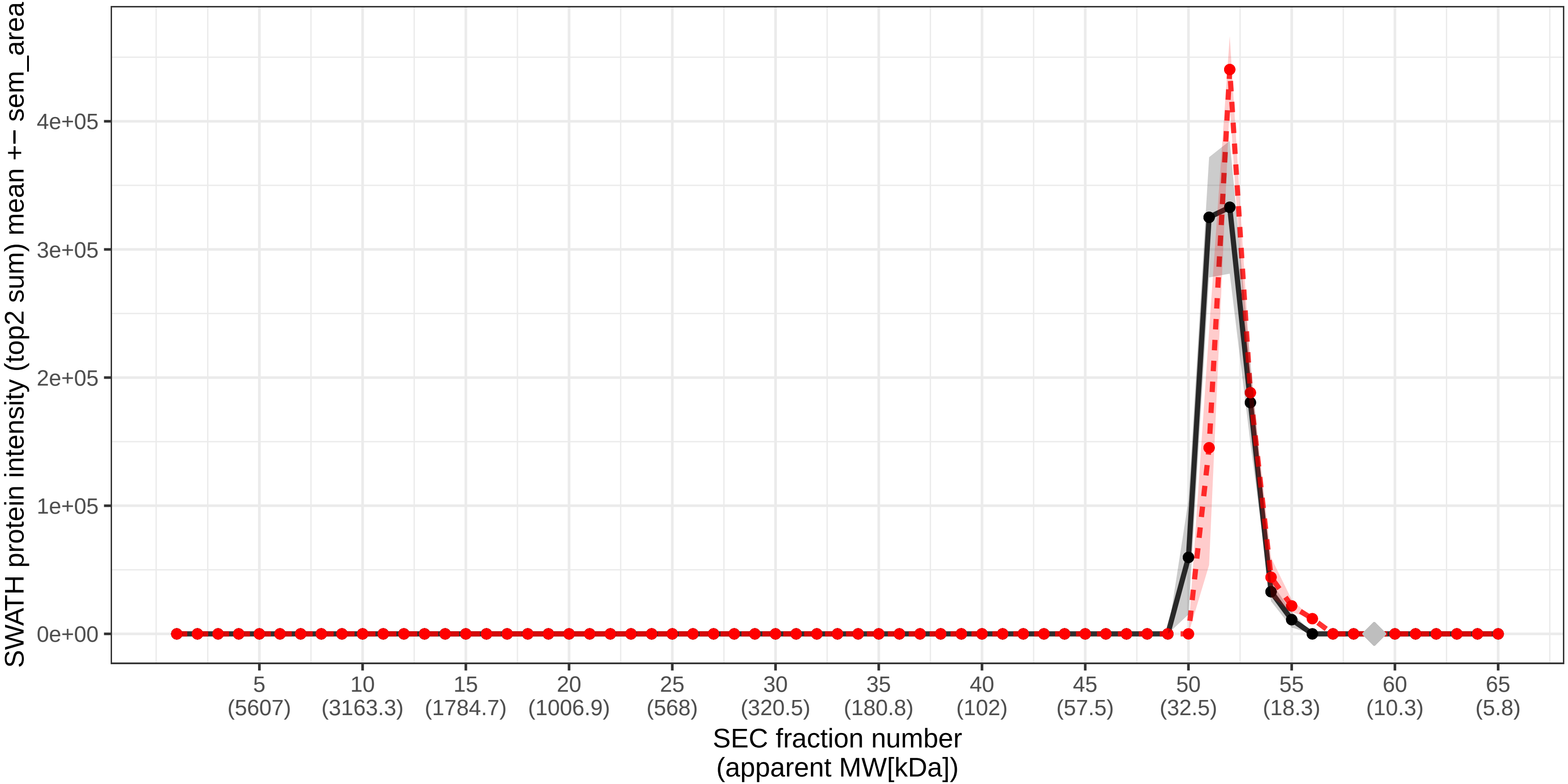

Supplement: Data S1. SEC-SWATH-MS Protein Chromatograms, Related to Figure 1 [file mmc6.zip › SECchrom_P29034_S10A2_HUMAN_S100A2_S100L.pdf]
